# Research on Displacement Tracking Device Inside Hybrid Materials Based on Electromagnetic Induction Principle

**DOI:** 10.3390/s25165143

**Published:** 2025-08-19

**Authors:** Xiansheng Sun, Yixuan Wang, Yu Chen, Mingyue Cao, Changhong Zhou

**Affiliations:** 1 China First Highway Engineering Co., Ltd., Beijing 100024, China; steve@phantomwhale.com; 2 Department of Architecture and Civil Engineering, City University of Hong Kong, Hong Kong SAR 999077, China; yxwang93-c@my.cityu.edu.hk; 3 Department of Architecture and Transportation Engineering, Guilin University of Electronic Technology, Guilin 541004, China; 18335094133@163.com (Y.C.); mycao@mails.guet.edu.cn (M.C.); 4 Guangxi Key Laboratory of ITS, Guilin University of Electronic Technology, Guilin 541004, China; 5 Key Laboratory of New Infrastructure Construction in the Transport Sector, Guilin University of Electronic Technology, Education Department of Guangxi Zhuang Autonomous Region, Guilin 541004, China

**Keywords:** particle-reinforced composites, electromagnetic induction, metal particles, coil sensors, finite element simulation

## Abstract

Magnetic induction imaging technology, as a non-invasive detection method based on the principle of electromagnetic induction, has a wide range of applications in the field of materials science and engineering with the advantages of no radiation and fast imaging. However, it has not been improved to address the problems of high contact measurement interference and low spatial resolution of traditional strain detection methods in bulk materials engineering. For this reason, this study proposes a magnetic induction detection technique incorporating metal particle assistance and designs a hardware detection system based on an eight-coil sensor to improve the sensitivity and accuracy of strain detection. Through finite element simulation and an image reconstruction algorithm, the conductivity distribution reconstruction was realized. Taking asphalt concrete as the research object, particle-reinforced composite specimens with added metal particles were prepared. On this basis, a hardware detection system with eight-coil sensors was designed and constructed, and the functionality and stability of the system were verified. Using finite element analysis technology, two-dimensional and three-dimensional simulation models were established to focus on analyzing the effects of different coil turns and excitation parameters on the induced voltage signal. The method proposed in this study provides a new technical approach for non-contact strain detection in road engineering and can also be applied to other composite materials.

## 1. Introduction

Particle-reinforced composites (PRCs) are widely used in all kinds of engineering structures, and it is especially important to obtain the internal stress–strain state and evolution law of such materials to reduce the occurrence of their early disease and improve the working performance so as to prolong the service life of engineering structures. Therefore, it is imperative to develop a fast and effective internal strain detection technology for particle-reinforced composites. Due to the great difficulty of micro-observation, analysis, and modeling of this material, traditional methods such as ground-penetrating radar [1], ultrasonic detection, and CT detection [2] have limited means of tracking and detecting and measuring the internal micro-view stress–strain state of the material. Magnetic induction tomography (MIT) [3], as an imaging technique to reconstruct the conductivity distribution of the object under test, is non-destructive, low-cost, and fast imaging and can reconstruct the conductivity distribution of the object by applying an excitation magnetic field to the measured space without damaging the structure of the object to be measured [4]. It has been widely used in the fields of medical image detection, industrial engineering, material science, metal flaw detection, etc. [5]. In this paper, we will discuss the basic method of obtaining the particle displacement inside the composite material and the formation of its device based on this technique.

MIT technology has been gradually developed from the end of the 20th century to the beginning of the 21st century. In 1993, Alzeibak and Saunder [6] first proposed the application of MIT to human body detection. Griffiths et al. [7] deduced the relationship between sensitivity and frequency by combining with the electromagnetic field theory and set up a single-channel induction system of 10 MHz, but the reconstruction effect is not good due to the complexity of the noise and image reconstruction algorithms. Park [8] used high-sensitivity lock-in amplifier and multi-channel simultaneous detection technology and improved the signal-to-noise ratio via an adaptive calibration algorithm to realize real-time imaging of flowing conductive fluids, which can be realized in real-time detection in industrial inspection. Chen and Vauhkone [9] verified the feasibility of MIT in detecting the anomalies of electrical conductivity by simulating different bleeding scenarios through Comsol simulation. Gursoy et al. [10] proposed a correction method for the sensor model error, which reduces the artifacts through accurate calibration and adaptive algorithms. Li et al. [11] proposed a linear approximation 3D imaging algorithm, which accelerates the orthogonal computation through one-dimensional Fourier transform, realizes fast and low-computation-volume full-space 3D imaging, and verifies its validity in the detection of buried metal pipes. These studies have promoted the application and development of MIT technology in the medical and industrial fields. However, image reconstruction algorithms and imaging efficiency are the biggest bottlenecks hindering the further development and application of this technology.

With the rapid development of computer technology, signal processing technology, sensor technology, and so on, magnetic induction imaging technology has achieved significant improvement in algorithm optimization and imaging quality. In magnetic induction imaging, Al-Zeibak and Saunder [6] firstly used the filtered inverse projection algorithm to reconstruct images of internal organs of the human body. Ma et al. [12] improved the image reconstruction method and proposed an iterative algorithm based on the synchronous iterative reconstruction technique and a non-iterative algorithm based on the Tikhonov regularization and singular value decomposition, which enhanced the imaging quality of high-conductivity targets such as copper and aluminum through multi-channel excitation, and aluminum and other high-conductivity targets were imaged. Wei et al. [13] proposed a flow parameter reconstruction method based on electromagnetic induction for low-conductivity two-phase flow imaging, and the imaging error was lower than 8% when the flow rate was stable. In 2016, Dekdouk et al. [14] further optimized the imaging of low-conductivity targets by proposing a nonlinear reconstruction algorithm, and through the model of an insulation-defective conductor and the experiments of a plastic foreign body in a conductive gel, they verified that the nonlinear method has higher imaging accuracy than the linear algorithm. The development of MIT technology has benefited from algorithmic innovations, from the early filtered inverse projection to iterative and non-iterative optimization to nonlinear reconstruction methods, which have gradually improved the imaging capability of targets with different conductivities and have demonstrated the potential for wider applications in medical and industrial inspections, especially for internal stress–strain measurements in materials and other aspects.

For the field of strain detection, various advanced methods have been proposed to improve the accuracy of defect identification and stress measurement. Wu [15] proposed a piling defect recognition method based on TC-CNN and wavelet packet denoising, fusing one-dimensional time-domain signals and two-dimensional time–frequency maps to achieve high-precision multi-defect classification. Ji [16] et al. used deep neural networks and 12 strain gauge sensors to achieve the four-point bending test to achieve accurate detection and localization of cracks in composite materials. An et al. [17] developed a stress detection device for steel structures based on the eddy current effect, which can quantify the strain changes during the elastic phase. Strain detection technology is developing towards multimodal, intelligent, and high precision, combining numerical simulation, artificial intelligence (e.g., neural networks), the eddy current effect, and optical sensing, which significantly improves the ability of structural health monitoring and defect identification and is suitable for industrial inspection and other fields.

**Figure 1:**
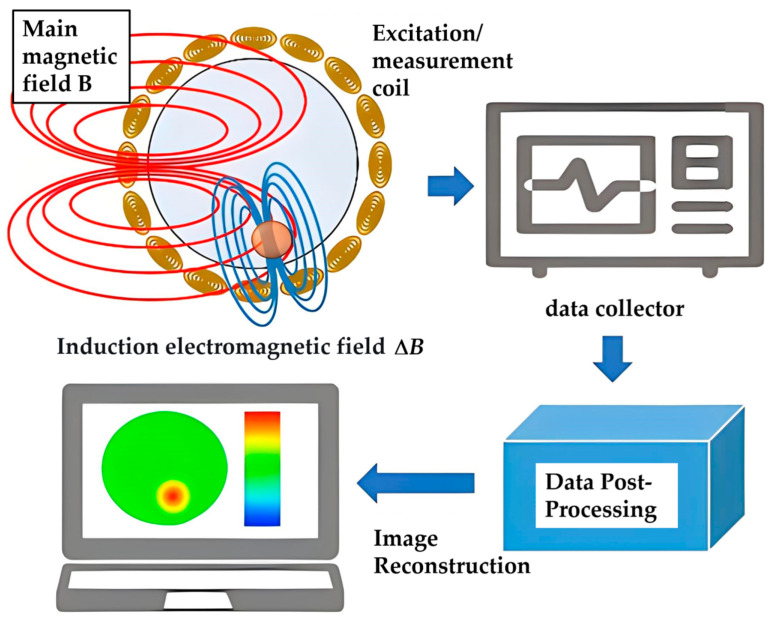
Typical MIT process.

MIT is a novel imaging technique that uses the conductivity distribution of the object under test as the imaging target, being a relatively new medical imaging technique with a typical imaging process, as shown in Figure 1. It serves as a typical method of non-contact detection, capable of penetrating deep into the material and reflecting the distribution of materials with different conductivities (resistivities). The application of this technique to the measurement of internal strain in PRCs can realize both the internal strain reconstruction of the material and the detection test of the microscopic properties of hybrid materials, which can undoubtedly greatly promote the in-depth study of this method.

However, since the MIT technology is just emerging and still in the state of research, traditional MIT imaging obviously carries the same disadvantage as CT detection: time consuming. However, PRCs are different from biological tissues or other homogeneous materials; on the one hand, it is not necessary to detect the conductivity distribution on each complete section, as we only need a certain number of values on the nodes, and we can complete the inversion of stress–strain (i.e., we can relax the technical requirements for imaging). On the other hand, particle-reinforced composites (such as asphalt mix or cement concrete) are a kind of mixed materials that need to be formulated (i.e., mixed materials have natural advantages for this kind of method), and it is entirely possible to add a small amount of additional special materials to improve the detection accuracy by improving the material resistivity contrast (i.e., with the potential of obtaining a higher accuracy by optimizing the design). This particular advantage of PRCs provides a favorable opportunity to improve the MIT reconfiguration technique and to develop a fast microfabricated stress–strain testing system.

In view of this, this paper takes an asphalt mixture as an example, introduces magnetic induction imaging technology into the field of internal structural strain detection of PRCs, builds a hardware imaging device, takes advantage of its non-contact, non-invasive, and high-sensitivity characteristics, and combines it with the multi-field coupling model to construct a strain distribution visualization method for PRCs, aiming to break through the bottleneck of the traditional detection technology on the characterization of internal strain evolution of the material and provide a new technical path for engineering materials’ detection.

Specifically, we make the following contributions:

(1) Based on the construction of a magnetic induction hardware detection system, this paper establishes a two-dimensional electromagnetic induction detection simulation model, which reveals the quantitative correlation between the electromagnetic parameters and the sensitive field in the MIT system and provides theoretical support for the inverse problem solving and the optimization of the actual system device.
(2) The raw material selection process was designed, and asphalt concrete specimens were prepared. By comparing the gap between the materials in terms of their order of magnitude and performance, it was concluded that the highly conductive properties of metals can sensitively reflect the internal strain of asphalt concrete, thus verifying the feasibility of metal particles as strain-assisting particles.
(3) The sensitivity matrix was constructed by electromagnetic field simulation. The image reconstruction was carried out by combining LBP, Tikhonov regularization, and the Landweber algorithm, and the imaging error was less than 15%. Experiments show that the system can detect the internal strain of PRCs quickly and effectively.

The rest of the paper is organized as follows: Section 2 describes the electromagnetic induction position system (eMIP) framework. Section 3 describes the design and construction of the electromagnetic induction displacement detection system. Section 4 presents the simulation study of the inverse problem of the electromagnetic induction displacement detection technique. Section 5 gives the experimental results and analysis. Section 6 derives the results and outlook.

## 2. Electromagnetic Induction Position System Framework

### 2.1. Faraday’s Law of Electromagnetic Induction

The derivative of the magnetic flux through the circuit with respect to time is known as the induced electromotive force and is also known as Faraday’s law of electromagnetic induction [18], which can be expressed as Equation (1):

(1) $$\varepsilon =-\frac{d\phi_{B}}{dt}$$

where ε is the induced electromotive force, $\phi_{B}$ is the magnetic flux in the system loop, and $d\phi_{B}/dt$ is the derivative of the flux with respect to time, where the negative sign denotes the corrugated law. In the MIT system, a time-varying magnetic field is generated by passing an alternating current through the excitation coil, which induces closed eddy currents in the conducting medium under test. The distribution of these eddy currents is directly related to the conductivity and provides a theoretical basis for conductivity reconstruction. The secondary magnetic field generated by the eddy currents is superimposed on the primary magnetic field, causing an interference to the initial primary field. The coupling interference of the primary magnetic field is suppressed by capturing the generated induced voltage through a sensor. The dynamic nature of Faraday’s law allows the MIT system to capture transient changes in conductivity and combine it with image reconstruction algorithms for industrial online monitoring or dynamic tracking of biological tissue [19].

### 2.2. Maxwell’s System of Equations

The physical basis of electromagnetic induction displacement detection is the principle of electromagnetic induction, which relies on the phenomenon of a conductor generating an electric potential in response to a varying magnetic flux and reconstructs the distribution of conductivity inside the object by detecting the magnetic field generated around the object under test as a function of time [20]. From the principle of electromagnetic induction, a set of Maxwell’s equations describing the harmonic variations of the MIT system is obtained:

(2) $$\left\{\begin{array}{l} \nabla \times \vec{H}=\sigma \vec{E}+j\omega t\vec{E}\\ \nabla \times \vec{E}=-j\omega \vec{B}\\ \nabla \cdot \vec{B}=0\\ \nabla \cdot \vec{D}=0 \end{array}\right.$$

The measured field is assumed to be linear, homogeneous, and isotropic, and the equation of the state is:

(3) $$\left\{\begin{array}{l} \vec{D}=\varepsilon \vec{E}\\ \vec{B}=\mu \vec{H}\\ \vec{J}=\sigma \vec{E} \end{array}\right.$$

where ε, μ, and σ denote the dielectric constant, magnetic permeability, and electrical conductivity of the material to be measured, respectively, and the above differential equations are in the international system of units. Bringing Equation (3) into the differential form of Maxwell’s system of equations, we have:

(4) $$\nabla \times \mu^{-1}\vec{B}=\sigma \vec{E}$$

We define the vector magnetic potential A such that it satisfies

(5) $$\nabla \times \vec{A}=\vec{B}$$

Combined with Equation (4), there is:

(6) $$\nabla \times \mu^{-1}\nabla \vec{A}=j\omega \sigma \vec{A}$$

According to the analysis of Equation (6), in the MIT system, auxiliary particles with different conductivities and permeabilities will affect the distribution of vector magnetic position *A*. By using these particles, the distribution of vector magnetic position *A* can be obtained.

### 2.3. System Architecture

**Figure 2:**
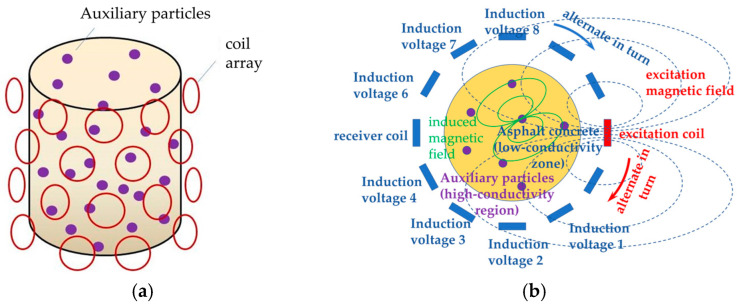
Schematic diagram of the principle of magnetic induction detection. (**a**) Schematic diagram of three-dimensional detection principle; (**b**) schematic diagram of two-dimensional detection principle.

The core idea and the most important innovation of this system is that a certain number of auxiliary particles (e.g., metallic materials) with significant differences in conductivity with non-metallic materials are added in advance during the mixing of the mixture, and in accordance with the theory of induced magnetic field, using the surrounding coil array, the localization and tracking of these particles are realized, and the displacement and strain field distribution on the micro- and fine scales of the materials are obtained by certain inverse algorithm reconstruction (Figure 2).

**Figure 3:**
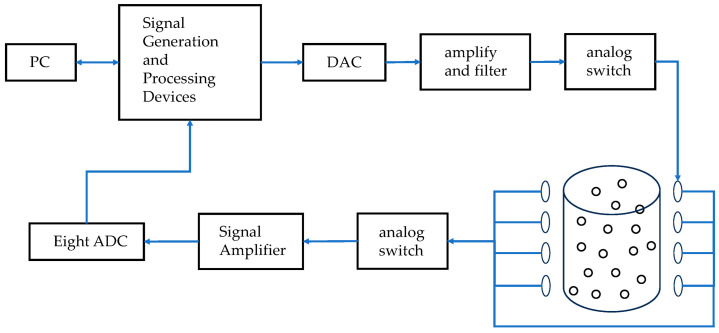
Hardware acquisition circuit.

Magnetic induction detection device hardware mainly includes the signal generation and processing device, signal excitation module, power amplification module, signal amplifier and signal acquisition module. The use of field-programmable gate array (FPGA) control generates sinusoidal excitation signals, and through the D/A conversion chip, the digital signal is converted to analog signals, and then sequentially through the amplification and filtering circuits and through the analog switch they can selectively pass into the excitation coil. Regarding metal auxiliary particles in the excitation of the magnetic field under the action of secondary magnetic field, the detection coil will be collected by the induction voltage signal into the analog switch, through the signal amplification, and finally through the eight-way A/D conversion module; it is then transferred to the host computer via USB for signal acquisition and analysis. The hardware acquisition circuit flowchart is shown in Figure 3.

## 3. Design and Construction of Electromagnetic Induction Displacement Detection System

Based on the aforementioned magnetic induction displacement detection architecture, this paper builds a hardware acquisition system based on 8 coils. And on this basis, a magnetic induction simulation model is established. Numerical calculation methods based on finite element analysis are used to study the electromagnetic induction displacement detection data selection mechanism, system sensitivity-influencing factors, and model optimization measures, and Comsol is used as the simulation modeling and analysis software to solve the measured values on the detection coil.

### 3.1. Particle-Reinforced Composites and Metal Property Analysis

To measure the internal strain displacement field and strain distribution of the specimen, auxiliary particles were incorporated in this study to facilitate the detection. Given the secondary role of these particles, excessive quantities may alter the intrinsic properties of the base material. Therefore, the number of particles should be minimized while still ensuring effective strain and displacement monitoring. This study recommends using no more than 10 particles, uniformly distributed throughout the specimen volume. The particle shape primarily influences eddy current generation, as complex geometries can complicate eddy current measurements. Experimental validation confirmed that spherical particles adequately meet measurement requirements; thus, this study primarily employs spherical particles.

We know that copper has higher electrical conductivity and a relative permittivity that is no lower than that of other metals. Based on these properties, we tested the relevant properties of copper and particle-reinforced composite materials at different frequencies to study the effect of embedded metal auxiliary particles on the electrical conductivity distribution of particle-reinforced composite materials. Copper has a magnetic permeability of 1, indicating that it is a weakly magnetic material with low magnetization in a magnetic field. Its relative permittivity ranges from 1 × 10^−7^–1 × 10^−8^, reflecting its relative ability to store electrical energy in an electric field.

(7) $$\delta =\sqrt{\frac{2}{\omega \mu \sigma }}=\sqrt{\frac{1}{\pi f\mu \sigma }}$$

Here, δ, f, ω, μ, and σ represent the skin depth, frequency, angular frequency, magnetic permeability, and electrical conductivity of the material, respectively. As shown in Equation (7), as the frequency increases, the skin depth decreases, meaning that at higher frequencies, the current flows more concentrated on the surface of the conductor.

PRCs have relatively low electrical conductivity, whereas metals usually have very high conductivity. With the exception of ferromagnetic materials, the permeability of metals is similar to that of vacuum. The dielectric constant of metallic materials is typically around 1. PRCs have a much higher dielectric constant. The skinning depth is used to measure the depth of electromagnetic wave penetration into the material, and the skinning depth of metals decreases gradually with the increase in frequency. Since most of the non-metallic PRCs are insulator materials [21] (e.g., the resistivity of asphalt concrete is about 10^7^~10^9^ Ωm) and the resistivity of metal is about 10^−8^~10^−7^ Ωm, the electrical conductivity of the metal particles is about 10^14^ times or more than that of the material under test, which is sufficient to accurately measure the secondary field. This is sufficient to accurately measure the small displacement changes of the particles and thus obtain the strain field distribution inside the material.

### 3.2. Hardware Device Design

**Figure 4:**
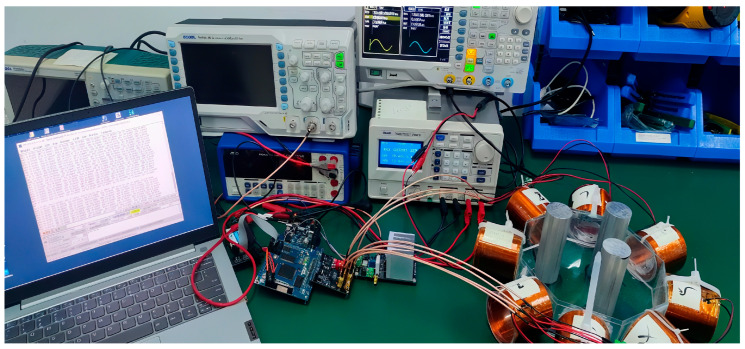
Electromagnetic induction displacement detection hardware system.

The magnetic induction detection device designed in this paper is shown in Figure 4, the hardware composition of the detection device is shown in Figure 3, and the principle of magnetic induction excitation and detection is shown in Figure 2b.

The device consists of eight coils, with one coil acting as the excitation coil and the remaining seven coils acting as induction coils. The 8 coils tested are rotated every 15°, obtaining 24 sets of test data and 192 induced voltage values. A steady AC current is applied to the excitation coil, which is excited to produce an alternating magnetic field. Under the action of the varying magnetic field, a varying secondary magnetic field is generated around the object under test. The magnetic field changes under the effect of the conductivity distribution of the object and is received using coil sensors distributed in the vicinity of the object under test. The sensor array consists of multiple detection coils (8 coils are used in this example), which allows for simultaneous acquisition of multiple data points and improves imaging speed and resolution. The signal acquisition unit is utilized to achieve signal front-end amplification, filtering, and conditioning of the weak signal output from the sensor. The detection coils capture changes in the magnetic field within the object field and are present in the coils in the form of electrical signals. The signal acquisition unit converts the analog signal into a digital signal and uploads it to the signal processing unit. The data processing and analyzing unit connects to the detection system through serial communication and uploads the information to the host computer. The physical diagram of the electromagnetic induction displacement detection hardware system is shown in Figure 4.

**Figure 5:**
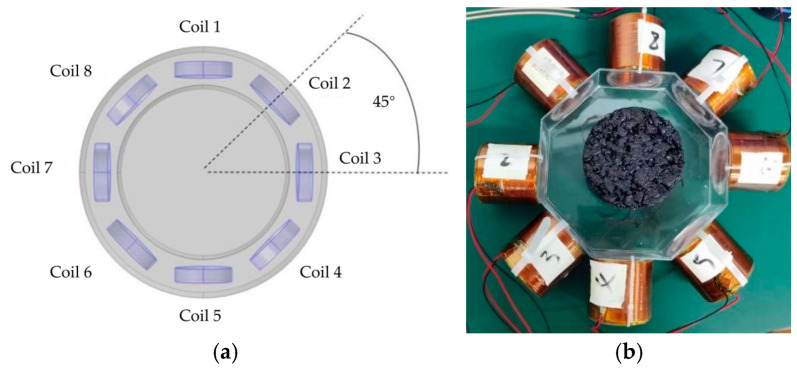
Coil sensor array distribution. (**a**) Distribution of sensor array; (**b**) physical drawing of coil.

As shown in Figure 5, the core of the model represents the object field environment, which is surrounded by eight identical induction coils uniformly distributed. A magnetic insulation layer is set between the magnetic field and the coils to effectively isolate the external interference and ensure the reliability of the detected signals. These coils are not only identical but also numbered in a clockwise direction from Coil 1 to Coil 8, maintaining a 45° space angle between each coil.

In this study, the selected coils are all copper enameled wire coils with a 1.2 mm wire diameter, as shown in Figure 5b, the center of which is the particle-reinforced composite material specimen to be tested. In order to ensure the consistency of the performance of the coils, the coil inductance is measured by using the IPT1000 series of inductance testers at 100 kHz, the relative inductance error of the coils is measured to be no higher than 0.11%, and the relative error of the coil Q value is measured to be less than 0.52%, which ensures the stability of coil performance. Specific data, as shown in Table 1, prove the rationality and effectiveness of the coils selected in this study in the application of magnetic induction detection system.

## 4. Analysis of the Influence Parameters of Electromagnetic Induction Displacement Detection Technology

In the electromagnetic induction displacement detection technique, the measured signals contain important information such as amplitude and phase, and each of them shows various variations associated with the electrical properties of the measured object. A group of coils was taken as excitation and named as coil No. 1. The rest of the coils were arranged clockwise in order, and groups 2 to 8 were the probe coils, which only quantitatively analyzed the object field in this part, and thus, there was no need to apply periodic excitation to each coil. In this study, only the real component of the voltage data was retained. By varying the coil turns, excitation current, and excitation frequency, we systematically investigated the resulting changes in the real-part induced voltage across the coils. The hardware system actually adopts the excitation frequency of 100 kHz, and in order to be closer to the real experimental environment, the simulation adopts 50 kHz, 100 kHz, and 200 kHz. The changes in the induced voltage on the detecting coils under several auxiliary particles are investigated at the same time. Based on the above model, the conductivity imaging was simulated to explore the effects of different data combinations on the imaging effect, and finally, the parameters applicable to this system were derived.

### 4.1. Finite Element Analysis Methods

Due to the very low electrical conductivity of PRCs (e.g., asphalt mixture), there is a significant contrast with the added metal particles. Metal particles can affect the induction coil, while the PRC (e.g., asphalt mixture) portion has almost no effect on the induction coil, thus enabling inversion. Although asphalt mixtures and other such materials are heterogeneous and consist of irregularly shaped particles, this does not affect the inversion process, and the material can be effectively treated as homogeneous. Since this study represents an initial-stage investigation and 3D modeling introduces significant complexity, a 2D approach was adopted to simplify the computational analysis.

**Figure 6:**
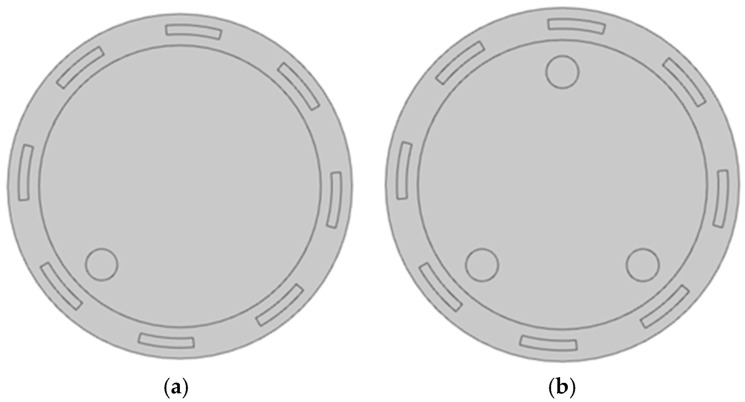
The 2D planar model: (**a**) 1 metal particle; (**b**) 2 metal particles.

The magnetic physical field was selected in Comsol. The geometrical model was built according to the actual detection system; the coils were 10.3 cm from the center of the physical field with a sector angle of 20°, and all coils were simplified to a homogeneous multi-turn current-carrying curve with an initial setting of 100 turns. The inner radius of the magnetic insulation layer is 9 cm, and the outer radius is 11 cm. The relative magnetic permeability of the air domain is set to 1 and the electrical conductivity to 1. The electrical conductivity of the intermediate metal auxiliary particles is set to 0, and the magnetic permeability is set to 1. One and three metal spherical objects were placed in the test field to simulate metal particles as the research objects. The established two-dimensional model is shown in Figure 6.

**Figure 7:**
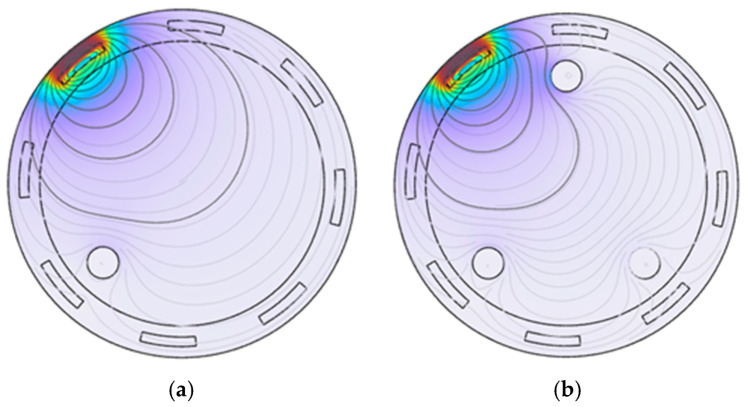
Magnetic flux density distribution: (**a**) 1 metal particle; (**b**) 2 metal particles.

To visually observe the magnitude and direction of magnetic flux density in the object field, Comsol was used to display the magnetic flux density distribution in a visual form using a cloud map. By analyzing these distributions, the relationship between the magnetic field and the electrical properties of the measured object can be understood. The first coil was used as the excitation coil, with an excitation frequency of 100 kHz and 100 coil turns. The cloud map is shown in Figure 7.

In Figure 7, it shows that there is an obvious area of high magnetic flux density with a color close to deep red near the excitation coil, and spreading outward from the excitation coil, the magnetic flux density gradually decreases and the color gradually becomes lighter, showing a recession trend. The flux density contours near the metal particles show obvious distortion and aggregation, which indicates that the metal particles have a significant effect on the magnetic field. In summary, metal particles have a significant effect on the magnetic flux density distribution, and information about the properties of metal particles can be obtained by indirectly analyzing these effects on the induced voltage of the surrounding detection coil.

### 4.2. Simulation Study of Coil Turn Influence Factor

**Figure 8:**
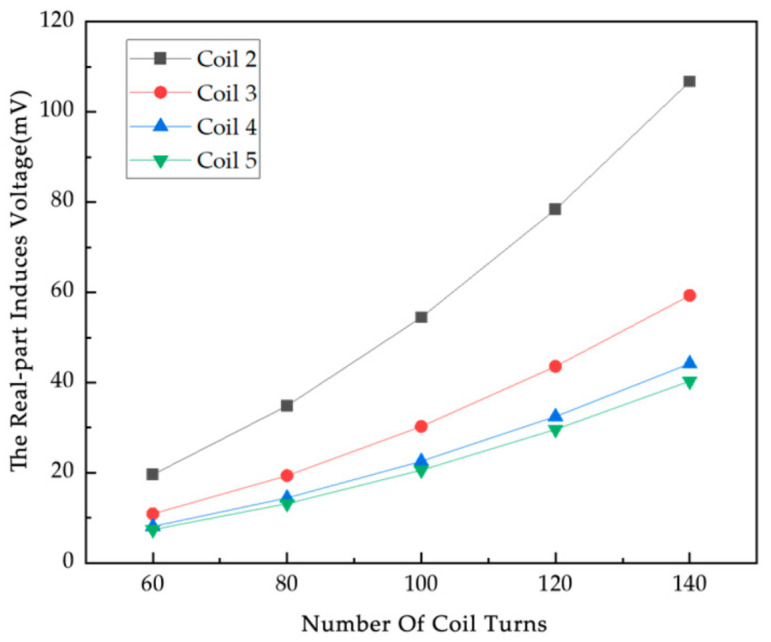
The real part of the induced voltage in each coil of the empty field versus the number of turns of the coil.

The number of coil turns is a key factor affecting the performance of electromagnetic induction displacement detection systems. A simulation study is carried out to investigate how the change in the number of coil turns affects the induced voltage of the system to provide a basis for optimizing the coil design. In this experiment, the range of values of the number of turns of the coil studied are determined to be 60 turns, 80 turns, 100 turns, 120 turns, and 140 turns. The excitation signal is 100 kHz/A. According to the law of electromagnetic induction, the induced voltage V in the detection coil is calculated for different numbers of turns, and the induced voltage increases accordingly for the same rate of change of magnetic flux with an increase in the number of turns of the coil. The induced voltage data for different number of turns of coil are obtained by simulation (Figure 8).

**Figure 9:**
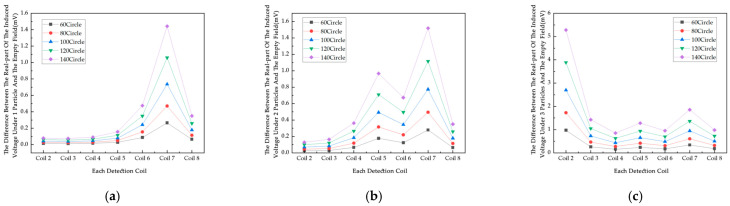
Variation in induced voltage of each coil with respect to the difference in the empty field under each metal particle: (**a**) 1 metal particle; (**b**) 2 metal particles; (**c**) 3 metal particles.

From Figure 8, it can be seen that the real part of the induced voltage on the coils from 2 to 5 under the empty field has the same trend of change with the increase in the number of turns, showing a monotonous increment, which is in accordance with Faraday’s law of electromagnetic induction. Figure 9 shows the difference between the induced voltage in the null field and the induced voltage on each coil with different numbers of particles, and the position of the particles is shown in Figure 7; from the data, the difference is all positive, which is the metal particles in the excitation of the magnetic field, due to the eddy current effect to cancel the strength of the original magnetic field. The induced voltage real and null field difference generally tends to increase with the increase in the number of turns of the coil. The growth rate of the voltage real part and empty field difference varies for different numbers of metal particles, and the induced voltage real part and empty field difference is higher and grows at a relatively fast rate under all coils when the number of coil turns is 140. From Coil 2 to Coil 8, the real part of the induced voltage and the difference from the empty field voltage increase to varying degrees under each metal particle, with the growth rate differing depending on the number of coil turns. At Coil 7, there is a significant peak in the real part of the voltage and the empty field difference at different numbers of particles, and the induced voltage difference in the real part of the coils at the same number of coil turns is different and non-monotonically varying, which is also determined by the position of the excitation coil and the metal particles. With more turns of the coil, the greater the resistance, affecting the sensitivity of the system, in order to facilitate the collection of data, so the number of turns of the coil used in this experiment is a compromise of 100 turns.

### 4.3. Simulation Study of Excitation Current Influence Factor

**Figure 10:**
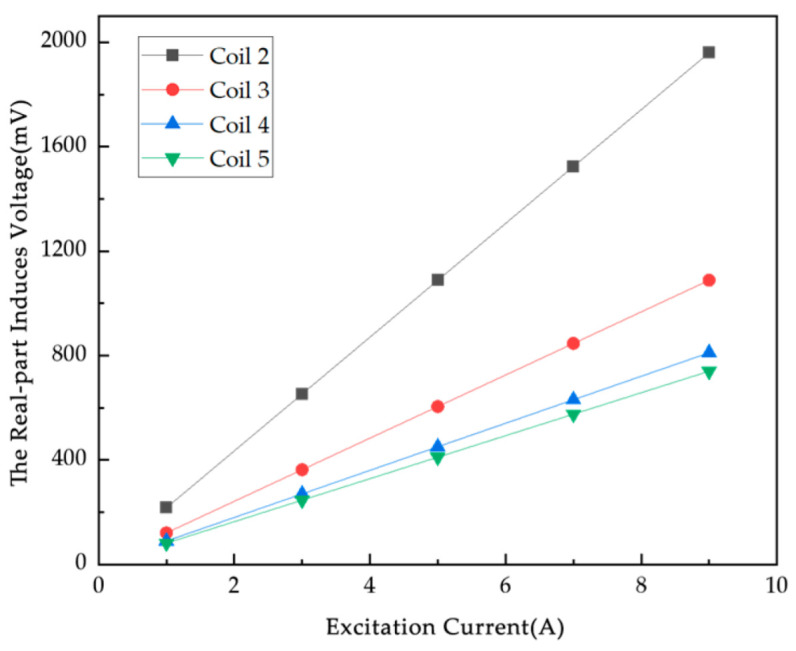
The real part of the induced voltage of each coil of the empty field versus the excitation current.

On this basis, a DC power supply based on AC voltage is designed. Normally, for a certain length of the straight conductor with current carrying, it is decomposed into a number of current elements by the Biot–Savart law, and then the total electric field is found by vector iteration. In this experiment, an AC signal is used for excitation, and the excitation currents are 1A, 3A, 5A, 7A, and 9A (Figure 10). The excitation frequency is 100 kHz for the magnetic field generated by the current-carrying wire when the excitation current increases, and the magnetic field strength and magnetic flux density will increase accordingly. The induced voltage data at different excitation currents are obtained by simulation.

**Figure 11:**
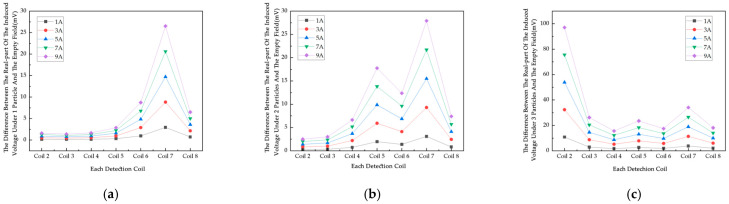
The change in real part of induced voltage difference between metal particles and empty field for various metal particles: (**a**) 1 metal particle; (**b**) 2 metal particles; (**c**) 3 metal particles.

In Figure 10, the real part of the induced voltage on Coils 2 to 5 in the empty field exhibits the same trend of monotonically increasing with increasing excitation frequency. The real part of the induced voltage and the empty field difference generally show an upward trend with increasing excitation frequency in Figure 11. The induced voltage real part of different coils under the same excitation current is different, and the change in the induced voltage difference of each coil is affected by the position of the particles and converges to the change under the excitation current. Most of the induced voltage difference in the real part is in the range of 0~15 mV under excitation frequencies of 50 kHz, 100 kHz, and 150 kHz, and the change is less obvious. The results show that for the conductive magnet, the frequency change has little effect on the measured results. Combined with the 200KSPS sampling rate of the chip AD7606 in the actual detection system, an excitation frequency of 100 kHz is used.

### 4.4. Simulation Study of Excitation Frequency Impact Factor

**Figure 12:**
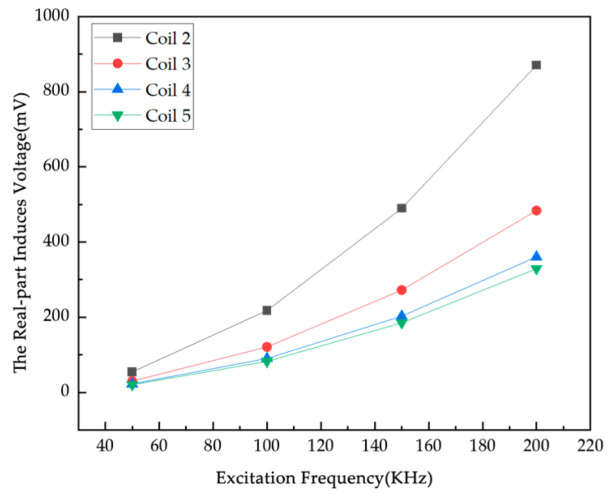
The real part of the induced voltage of each coil with the excitation frequency under an empty field.

Combined with the actual application scenario of the system, this experiment uses a sinusoidal AC signal for excitation, the excitation current is 1A, and four frequencies, 50 kHz, 100 kHz, 150 kHz, and 200 kHz, are used for simulation (Figure 12). According to the law of electromagnetic induction, the induced voltage in the detection coil is calculated under different excitation frequencies. Since the excitation frequency directly determines the rate of change of magnetic flux, an increase in frequency increases the flux change, and the induced voltage fluctuates significantly. On the one hand, a higher excitation frequency may enhance the penetration ability of the magnetic field, which helps to improve the detection ability of deep objects; on the other hand, too high a frequency may lead to increased electromagnetic interference, signal attenuation, and other problems, thus reducing the imaging resolution and sensitivity. The induced voltage values at each excitation frequency are obtained through simulation.

**Figure 13:**
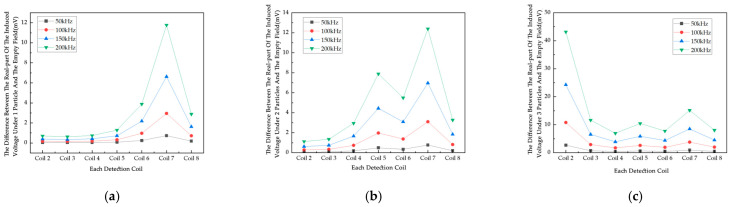
Variation in the real component of induced voltage difference across measurement coils (**a**) 1 metal particle; (**b**) 2 metal particles; (**c**) 3 metal particles.

As shown in Figure 12, the real part of the induced voltage on Coils 2 to 5 in the empty field exhibits the same trend of increasing monotonically with an increase in excitation current. As shown in Figure 13, with the increase in excitation current, the real part of the induced voltage and the empty field difference generally show an upward trend. The real part of the induced voltage differs among different coils under the same excitation current. In Figure 13c, the induced voltage difference of Coil 2 is relatively high, which is due to the influence of the position of the third particle. Since the actual coil wire diameter is 1.2 mm, the maximum current that can be passed through it is calculated to be 1 A. The excitation current needs to take into account the current-carrying capacity of the wire, so an excitation current of 1 A was used in this experiment.

### 4.5. Simulation Study on the Influence Factor of Conductivity of Metal Particles

**Figure 14:**
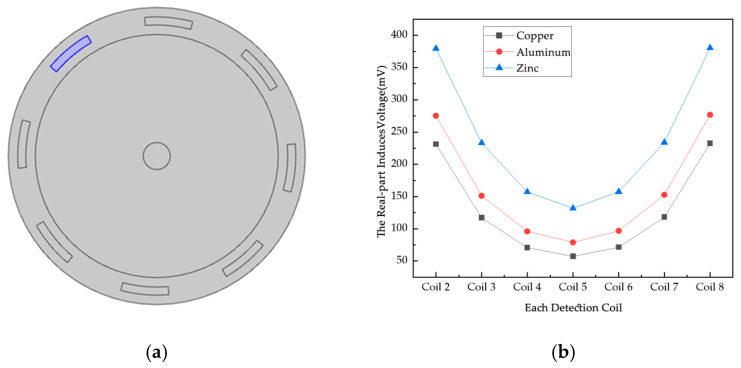
Effect of different materials on induced voltage. (**a**) Schematic diagram of the location of metal particles; (**b**) results of induced voltage test on different metal solid parts.

For metal particles, a higher conductivity means a greater number and mobility of free electrons within them. When an applied electric field is applied to metal particles, particles with high conductivity are able to move electrons from one position to another more efficiently, resulting in a larger induced current. The skin effect is more pronounced for metal particles with high conductivity. The high-conductivity metal particles, due to their good electrical conductivity, make it easier for the current to flow on the surface, which further enhances the strength of the induced current, resulting in a change in the magnetic flux through the metal particles. According to Faraday’s law of electromagnetic induction, the change in magnetic flux generates an induced electromotive force in the auxiliary particles, which, in turn, drives the generation of an induced current. Metal particles with high conductivity are able to change the magnetic flux more efficiently due to their larger induced currents, thus having a greater effect on the external magnetic field. In this section, metallic spherical particles made of three materials, copper, aluminum, and zinc, are used to test the effect on the induced voltage. The excitation signal is 100 kHz/A. The results of the comparison are shown in Figure 14.

As shown in Figure 14a, the metal particles are placed in the middle of the object field. As can be seen from Figure 14b, when the particles are located in the middle of the magnetic field, the induced voltage on each detection coil is different and is related to the position of the detection coil with respect to the excitation coil; the larger the distance, the smaller the magnetic field density, and the smaller the value of the induced voltage. The induced voltage of the coils is greatest when zinc is the metal particle. The conductivity of copper is about 5.998 × 10^7^ S/m, aluminum is about 3.774 × 10^7^ S/m, and zinc is about 1.69 × 10^7^ S/m. The higher the conductivity of the particles, the lower the value of the voltage on the corresponding detecting coils, which is due to the fact that induced currents will be generated internally when the object to be measured is put into the alternating magnetic field. Due to the eddy current effect, the higher the conductivity of the metal particles, the larger the eddy current generated under the same alternating magnetic field, and the greater the obstruction to the original magnetic field.

## 5. Inversion of Electromagnetic Induction Displacement

### 5.1. MIT Inverse Problem Theory

Based on the induced voltage signals of each coil of the object field obtained by solving the forward problem, the distribution information of the electric conductivity σ and magnetic permeability μ of the object is solved by Comsol with Matlab co-simulation. Based on Maxwell’s equations, the positive problem model combines the geometry of the target object and the electromagnetic properties of the material to illustrate the generation mechanism of the magnetic field or induced electromotive force at the external measurement points under the excitation of the applied magnetic field; the inverse problem model uses the measurement data as the input to solve the distribution of the internal conductivity, magnetic permeability, and other parameters, which is faced with serious unsuitability and needs to be transformed into a solvable form with the aid of regularization and optimization algorithms. The arithmetic description of the inverse problem reflecting the MIT technology in a two-dimensional object field space is shown in Equation (8):

(8) $$\left\{\begin{array}{l} \sigma \left(x,y\right)=f\left(G_{\sigma }\left(B_{ex}\left(x,y\right),B_{obj}\left(x,y\right),\sigma \left(x,y\right),\mu \left(x,y\right),\varepsilon \left(x,y\right)\right),V\right)\\ \mu \left(x,y\right)=f\left(G_{\mu }\left(B_{ex}\left(x,y\right),B_{obj}\left(x,y\right),\sigma \left(x,y\right),\mu \left(x,y\right),\varepsilon \left(x,y\right)\right),V\right)\\ \varepsilon \left(x,y\right)=f\left(G_{\varepsilon }\left(B_{ex}\left(x,y\right),B_{obj}\left(x,y\right),\sigma \left(x,y\right),\mu \left(x,y\right),\varepsilon \left(x,y\right)\right),V\right) \end{array}\right.$$

where $G_{\sigma }$, $G_{\mu }$, and $G_{\varepsilon }$ are the spatial sensitivities of the conductivity, magnetic permeability, and dielectric constant in the object field obtained by solving the positive problem, respectively. In MIT, *B_ex_* (experimental data) denotes the coil-induced voltages measured by sensors, serving as the input to the inverse problem. On the other hand, *B_obj_* (simulated data) represents the numerically computed magnetic field data obtained from the forward solution. During the iterative reconstruction process, the conductivity distribution σ is iteratively adjusted to minimize the discrepancy between $B_{obj}$ and $B_{ex}$. It can be seen that the solution of the MIT inverse problem is based on the known detection voltage *V*, solving the spatial distribution of the binary functions of conductivity, permeability, and dielectric constant.

### 5.2. Modeling the Sensitivity Matrix

The anisotropic regime of magnetism is called a soft magnetic field, and its susceptibility matrix varies nonlinearly with the distribution of the dielectric constant of the medium. At the same time, some mutual exclusion phenomena occur during the solution of the inverse problem. To solve this problem, the distribution law of dielectric constant needs to be understood firstly, and the solution of the sensitivity matrix is the key to realize the image reconstruction [22]. In addition, the sensitivity matrix and the distribution of dielectric constant are characterized by a nonlinear relationship. The induced voltage in the detection coil exhibits a nonlinear response to conductivity perturbations, where local variations in conductivity Δσ produce disproportionate changes in the measured voltage signal; the value of Δσ in the actual object field is small, and when the perturbation occurs as $\sigma^{\prime }=\sigma +\Delta \sigma$, the change in the voltage can be obtained as:

(9) $$\nabla V=\frac{df}{d\sigma }\Delta \sigma$$

where df/dσ is denoted as the matrix of coherence sensitivity coefficients, which is discretized:

(10) ∇V=SΔσ

where *S* is the m × n-dimensional sensitivity matrix in discrete form, m is the number of object field division cells, and n is the number of detected voltage signals; this experiment uses 64 × 64 sensitivity degree grid division. Setting 1 × n-dimensional detection signal change vector matrix as *e*, the conductivity distribution can be derived from *g*. The formula is deformed as in Equation (11), and the visual reconstruction of the conductivity distribution situation is achieved by establishing the relationship between the conductivity change, the voltage change, the measurement signal change, and the sensitivity matrix.

(11) $$g=S^{-1}e$$

### 5.3. Image Reconstruction Algorithms

#### 5.3.1. LBP Linear Backprojection Algorithm

The LBP algorithm is a non-iterative image reconstruction algorithm widely used in the field of tomography imaging. Its basic idea is to “back-project” the projection data obtained at different angles back to the imaging plane so as to obtain the reconstructed image. In the two-dimensional model, an object is ray-scanned at different angles, the projection data are obtained, and the points on the imaging plane correspond to different ray paths at different angles. The LBP algorithm back-projects the projection data at all angles to each point on the imaging plane according to the above relationship. However, due to the “soft field effect” in the object field, when the conductivity of the measurement area changes, it leads to the deformation of the sensitive field, thus affecting the accuracy of the conductivity reconstruction. In the actual discrete calculation, the integral of this reconstruction formula will become a summation form, and the formula will be converted to Equation (12) for the projection data under *N* angles:

(12) $$f\left(x,y\right)=\frac{1}{N}\sum_{i=1}^{N}p\left(x\cos\theta_{i}+y\sin\theta_{i},\theta_{i}\right)$$

In this paper, the electromagnetic induction displacement detection system uses a 64 × 64 mesh for the mesh division of the sensitivity, and the mesh spacing is calculated. In the inverse problem, the sensitivity matrix constructed with the finite element method itself is an irreversible matrix, which can be approximated by the transposed matrix approximate representation of the inverse matrix, whose expression is:

(13) $$G=S^{T}V$$

In Equation (13), *G* is the gray value of the object field, *S^T^* is the inverse matrix of the approximate sensitivity matrix, and *V* is the induced voltage.

#### 5.3.2. Tikhonov Regularization Algorithm

The Tikhonov regularization algorithm, a method for solving systems of pathological linear equations and preventing overfitting problems in regression analysis, is commonly used to solve ill-posed problems. Let a system of ill-posed linear equations be $F\left(x\right)=b$, where *F* is the linear operator, *x* is the solution sought, and *b* is the observed data. In some cases, insufficient observed data or differences in the number of divided grids and the detected data lead to an ill-posed system of equations (i.e., the matrix is not invertible), and a direct solution may produce oversized or unstable results. In order to obtain a stable solution, a regularization term *R*(*x*), which is usually a quadratic parameter of the solution, is introduced to construct the regularization problem, i.e., to solve the following minimization problem:

(14) $$R\left(x\right)=\min\left({\left|\alpha \right|}^{2}+\alpha {\left|Ix\right|}^{2}\right)$$

where α is the regularization parameter, *I* is the unit matrix, and $\left|Ix^{2}\right|$ is the regularization function. To solve for the minimal value of the regularization term *R*(*x*), let $\nabla R\left(x\right)=0$, that is:

(15) $$\nabla R\left(x\right)=F^{T}F-F^{T}b+\alpha I^{T}Ix=0$$

The desired solution can be obtained as:

(16) $$x={\left(F^{T}F+\alpha I\right)}^{-1}F^{T}b$$

The key to this solution lies in the value of the regularization parameter. Too large a value of α will lead to excessive smoothing, which makes the solution of the original equation have a large error; at this time, the regularization term is dominant, the ability of anti-noise interference is strong, and the solution of *x* tends to be close to 0. If the value of α is too small, it cannot effectively inhibit the ambient noise, and the solution is close to the original system of equations, with poor stability and a weak role of the regularization. In this experiment, the empirical value of α = 0.02 is used to calculate the solution *x*. This method effectively balances the weights of the data-fitting term and the regularization term.

#### 5.3.3. Landweber’s Algorithm

Landweber’s algorithm is an iterative algorithm for linear ill-posed problems. Landweber’s algorithm is able to suppress the effect of noise in the measurement data on the solution to a certain extent and obtain a more stable solution. For $F\left(x\right)=b$, the Landweber algorithm is an iterative algorithm for linear ill-posed problems, where *F* is the linear operator, *x* is the solution sought, and *b* is the observed data. When *F* is an ill-posed matrix, solving the equation directly will result in a solution that is very sensitive to noise in the measurement data. The solution is obtained by iterating through Landweber’s algorithm, which builds the objective function, optimizes it, and derives it:

(17) $$\nabla F\left(x\right)=F^{T}Fx-F^{T}b=F^{T}\left(F^{T}-b\right)$$

*F^T^* in Equation (17) is the transpose matrix of *F*. With the negative gradient direction as the optimization direction, the k+1st iteration of Equation (18) is:

(18) $$X_{k+1}=X_{k}+\omega F^{T}\left(b-Fx_{k}\right)$$

In the formula, ω is the relaxation factor, the size of which affects the stability of the solution, and ω has an important effect on the convergence of the algorithm and the convergence speed. If ω is too large, the algorithm may not converge; if ω is too small, the algorithm will converge very slowly. Theoretically, the value range of ω is generally $0<\omega <2/\alpha_{\max}$, and ω is the largest eigenvalue of *F^T^F*. In practice, we need to choose the appropriate ω to ensure the convergence of the algorithm through experiments. Set the maximum number of iterations *k* that terminate when the number of iterations reaches k, i.e., when $\left\|b-Fx_{k}\right\|<\varepsilon$, ε is the preset threshold.

### 5.4. Case Study

In this section, we conduct simulation arithmetic experimental analysis of the research model and use image reconstruction algorithms to analyze the distribution of the measured object’s permeability or conductivity in different directions as a means of verifying the feasibility of the model and the accuracy of the results.

**Figure 15:**
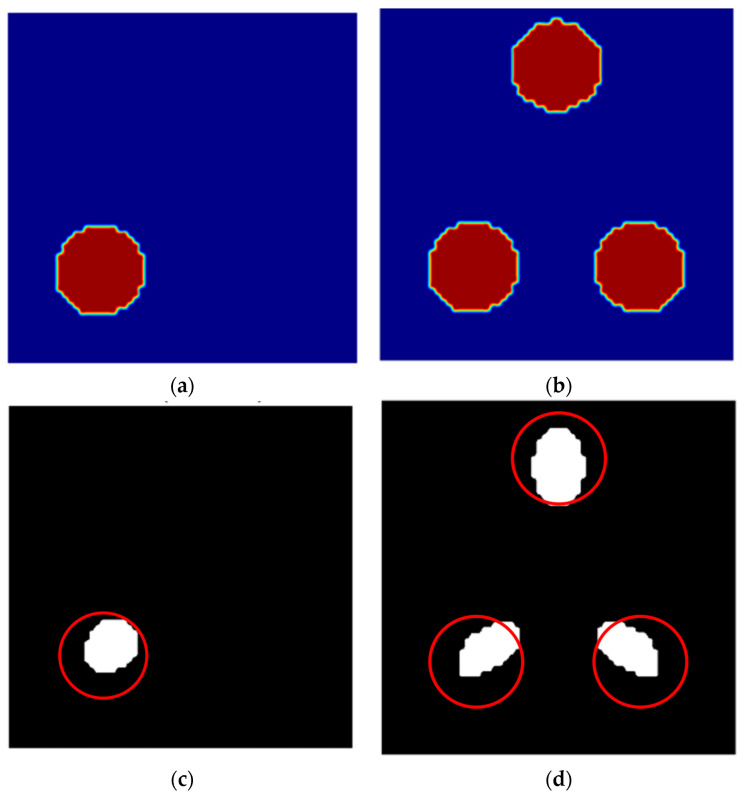

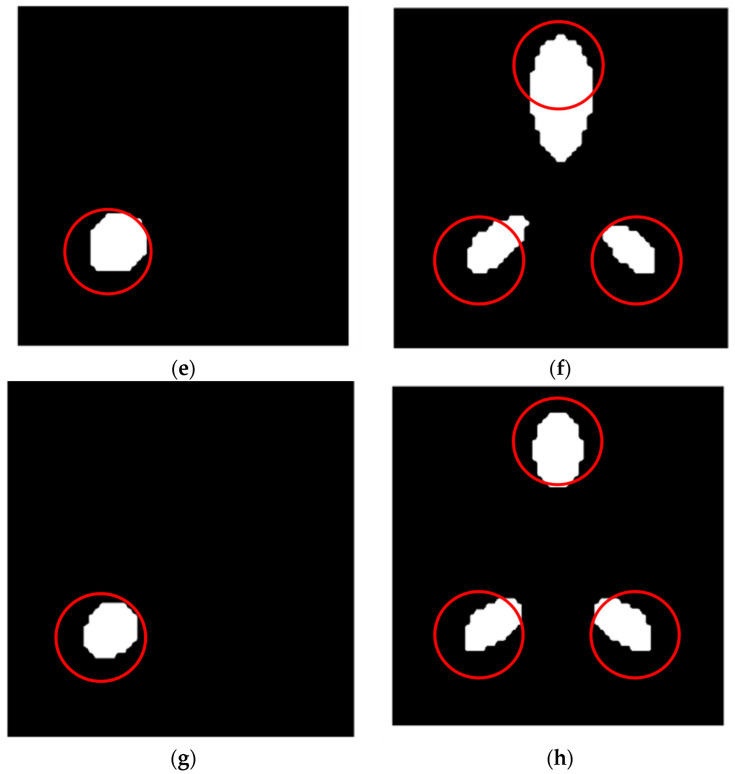
Results of comparative analysis of image reconstruction algorithms where the red circles indicate the locations and outlines of the actual metal auxiliary particles. (**a**,**b**) Standard type; (**c**,**d**) LBP; (**e**,**f**) Tikhonov; (**g**,**h**) Landweber.

Through joint simulation with Comsol and Matlab, based on the sensitivity and empty field and full field voltage solution results, the LBP linear inverse projection algorithm, Tikhonov regularization algorithm, and Landweber algorithm were further solved. Figure 15 shows the standard image type and the reconstructed image of the three imaging algorithms. The three algorithms in the figure correspond to different numbers of auxiliary particles (one and three).

The image of the LBP algorithm with one auxiliary particle is fuzzy, the shape and size are more approximate, and the strain distribution is not precise enough, while the images of the Tikhonov regularization and Landweber algorithms show a more concentrated region, which indicates that the strains are concentrated at a specific location, but the image of the Tikhonov regularization is slightly clearer. In the case of three auxiliary particles, the LBP algorithm’s image shows three regions, but the overall image is blurry and noisy; the Tikhonov regularization shows three clearer regions with better noise control, and the Landweber algorithm’s image shows three regions as well but is not as accurate as the Tikhonov.

A comparison between the true center coordinates of the particles and the inverted coordinates is presented in Table 2. This study compares the center coordinates of each particle in the standard type with the particle position coordinates inverted by the corresponding algorithm, using the mean absolute error (MAE) of the corresponding x- and y-axis coordinates as the evaluation criterion. It can be concluded that, in the case of one particle, the MAE values for the LBP algorithm, Tikhonov regularization, and Landweber algorithm are 0.165, 0.09, and 0.165, respectively. In the case of three particles, the MAE values for the LBP algorithm, Tikhonov regularization, and Landweber algorithm are 0.168, 0.368, and 0.182, respectively. It can be seen that in the case of a single particle, the three algorithms show little overall difference, but in the case of multiple particles, the accuracy of the LBP algorithm and Landweber algorithm is significantly higher than that of Tikhonov regularization. Furthermore, the study found that regardless of the algorithm used, the particle images obtained through inversion in the case of three particles are more elongated compared to the case of one particle, which is related to the mutual influence between particles under magnetic field conditions. This study concludes that while Tikhonov regularization performs best in single-particle scenarios, its accuracy is notably inferior to the other two algorithms in multi-particle scenarios. Therefore, the LBP algorithm and Landweber algorithm perform optimally in asphalt concrete strain detection based on electromagnetic induction displacement detection, particularly in scenarios with a larger number of auxiliary particles, providing clearer and more accurate strain distribution images.

## 6. Conclusions

In this paper, we have carried out a detailed study and analysis of the electromagnetic induction displacement detection theory, hardware imaging device design and implementation, electromagnetic induction displacement detection, positive and inverse problems, etc., and functionally verified the constructed hardware system, combined with the conductivity reconstruction algorithm to validate the practical value of utilizing metal particles as an auxiliary material, to realize the method of detecting the internal strain in the granular-reinforced composites. In this study, the use of metals as auxiliary particles in the magnetic induction detection system and the comparative analysis of the conductivities of common metals such as copper, aluminum, etc., and asphalt concrete differed by more than 10^10^ times in order of magnitude, illustrating the feasibility of using metals as auxiliary materials for strain detection, which can effectively capture the small displacement or deformation of metal particles and thus effectively assist in the inversion of strain field distribution inside particulate-reinforced composites.

This study mainly builds a magnetic induction hardware detection system, using CYCLONE IV EP4CE30 as the main control chip FPGA development platform, consisting of a minimum system, excitation module, signal acquisition module, and eight-coil sensor system. In this work, we measured the actual phase angle value of each coil and the demodulation of the phase angle value error within 0.3%, performing experiments on asphalt concrete specimens to verify the functionality and stability of the hardware system. Finally, the sensor coil is optimized and designed with a single layer of 100-turn copper enameled coil, which achieves stable excitation and acquisition effects. Respectively, we established the Comsol two-dimensional and three-dimensional simulation models; the simplified two-dimensional simulation model was for conductivity imaging-related data selection simulation analysis to obtain different coil turns, excitation currents, and excitation frequencies under the change rule of the induced voltage signal. At the same time, combined with the influence of hardware device parameters, it was decided to select 100 coil turns, 1A/100 kHz excitation as the best experimental parameters, and zinc as the optimal auxiliary material because it can effectively improve the sensitivity of the system. On this basis, the conductivity was reconstructed using the LBP linear inverse projection algorithm, Tikhonov regularization method, and Landweber algorithm to achieve higher-quality image reconstruction, and the reconstruction results were analyzed. The analysis determined the applicability of different algorithms under different conditions, laying the foundation for high-quality inversion in the later stages.

In the future, neural networks will be introduced to continue the in-depth exploration of inversion algorithms, with the aim of establishing a sound technical system and efficient detection devices. The size of auxiliary particles will be reduced, the number of particles increased, and the method will be extended to 3D, continuing research in a more practical direction.

## Figures and Tables

**Table 1:** Comparison of electrical characteristics of different coils.

Transformers	Inductance (μH)	Resistance (Ω)	Q Value (Inductive/Resistive)	Multimeter Electric (Ω)
1	452.94	0.39	7.27	0.37
2	461.81	0.39	7.35	0.39
3	459.06	0.38	7.28	0.39
4	460.08	0.39	7.31	0.38
5	463.61	0.39	7.36	0.39
6	459.11	0.38	7.25	0.36
7	460.12	0.39	7.33	0.38
8	462.66	0.39	7.36	0.39

**Table 2:** Data table of comparison of particle center position.

Number of Particles	Coordinate	Real Value	LBP	Tikhonov	Landweber	Error Relative to the True Value (cm)			MAE		
1 particle	x_1_	−5	−4.83	−4.91	−4.83	−0.17	−0.09	−0.17	0.165	0.090	0.165
	y_1_	−5	−4.84	−4.91	−4.84	−0.16	−0.09	−0.16			
3 particles	x_1_	−5	−4.78	−4.63	−4.76	−0.22	−0.37	−0.24	0.168	0.368	0.182
	y_1_	−5	−4.76	−4.64	−4.75	−0.24	−0.36	−0.25			
	x_2_	5	4.78	4.76	4.77	0.22	0.24	0.23			
	y_2_	−5	−4.84	−4.68	−4.81	−0.16	−0.32	−0.19			
	x_3_	0	−0.02	0.01	−0.01	0.02	−0.01	0.01			
	y_3_	6	5.85	5.09	5.83	0.15	0.91	0.17			

## Data Availability

Dataset available on request from the authors.

## Abbreviations

The following abbreviations are used in this manuscript:

MIT	Magnetic Induction Tomography
TC-CNN	Two-Channel Convolutional Neural Network
CT	Computed Tomography
LBP	Local Binary Pattern
eMIP	Electromagnetic Induction Position
FPGA	Field-Programmable Gate Array
MAE	Mean Absolute Error
